# Metastatic Breast Cancer Presenting as an Organizing Pneumonia Pattern on Chest CT: A Case Report

**DOI:** 10.7759/cureus.104358

**Published:** 2026-02-27

**Authors:** Ami Hirata-Matsuo, Toyoshi Yanagihara, Natsumi Kushima, Yoshihiro Hamada, Takato Ikeda, Masaki Fujita

**Affiliations:** 1 Department of Respiratory Medicine, Fukuoka University Hospital, Fukuoka, JPN; 2 Department of Pathology, Fukuoka University Faculty of Medicine, Fukuoka, JPN

**Keywords:** air-space consolidation, breast cancer, computed tomography, organizing pneumonia, pulmonary metastasis

## Abstract

Pulmonary metastasis from breast cancer typically presents as multiple well-defined nodules on chest imaging. Atypical radiological patterns, including air-space consolidation mimicking organizing pneumonia (OP), are exceedingly rare and diagnostically challenging. We report a case of a 68-year-old woman with a history of left breast cancer treated 26 years earlier who developed new bilateral bronchovascular bundle thickening with surrounding ground-glass opacities and consolidation on chest CT, mimicking OP. The patient had been receiving fulvestrant for known pulmonary metastasis diagnosed 14 years prior. A transbronchial biopsy from the left lower lobe revealed adenocarcinoma with positive immunostaining for estrogen receptor and GATA-binding protein 3, consistent with metastatic breast cancer. Treatment with S-1 resulted in radiological improvement at four months. This case adds to the limited literature documenting OP-mimicking pulmonary metastasis from breast cancer and highlights the importance of maintaining a high index of suspicion and pursuing tissue diagnosis in patients with a history of breast cancer who present with new pulmonary infiltrates.

## Introduction

Breast cancer is one of the most prevalent malignancies worldwide and a leading cause of cancer-related mortality among women. The lungs are among the most common sites of distant metastasis, with lung and/or pleural involvement reported in approximately 80% of women who died with metastatic breast cancer at autopsy [1]. The classic radiological presentation consists of multiple well-circumscribed round nodules distributed predominantly in the lung periphery [2]. However, pulmonary metastases from breast cancer can occasionally exhibit atypical imaging patterns, including solitary nodules, lymphangitic carcinomatosis, endobronchial metastasis, cavitary lesions, and air-space consolidation [2,3].

Among these atypical presentations, metastatic disease manifesting as air-space consolidation resembling organizing pneumonia (OP) is particularly rare. OP is characterized by patchy areas of consolidation, often with lower lobe predominance, ground-glass opacities (GGOs), and bronchovascular bundle thickening [4]. Because these radiological features significantly overlap with those of metastatic disease presenting as air-space consolidation, distinguishing between the two on imaging alone is often impossible [5,6].

To date, only a few case reports have described breast cancer pulmonary metastasis presenting with an OP-like pattern on chest CT [5-7]. The rarity of this presentation and the potential for misdiagnosis as cryptogenic OP (COP) or drug-induced pneumonitis highlight the importance of maintaining clinical vigilance and pursuing tissue diagnosis when appropriate.

Here, we report a case of a 68-year-old woman with a remote history of breast cancer who developed pulmonary metastasis presenting as bilateral OP-mimicking infiltrates on chest CT, confirmed by transbronchial biopsy, and successfully treated with S-1 chemotherapy.

## Case presentation

A 68-year-old Japanese woman presented with progressive dyspnea and cough. Her medical history was significant for left breast cancer diagnosed 26 years earlier, for which she had undergone left mastectomy with axillary lymph node dissection followed by adjuvant therapy. Fourteen years prior to the current presentation, she was diagnosed with pulmonary metastasis from breast cancer, and systemic therapy was initiated. Over the intervening years, she received various treatment regimens and had been receiving fulvestrant up to the time of the current admission.

Her medical history also included hypertension, type 2 diabetes mellitus, hyperlipidemia, and deep vein thrombosis. She was a lifelong nonsmoker with occasional alcohol use. Her family history was notable for gastric and colorectal cancer in her father. Her medications included insulin glargine, insulin lispro, rosuvastatin, famotidine, ursodeoxycholic acid, mecobalamin, and apixaban, in addition to fulvestrant for breast cancer.

**Figure 1:**
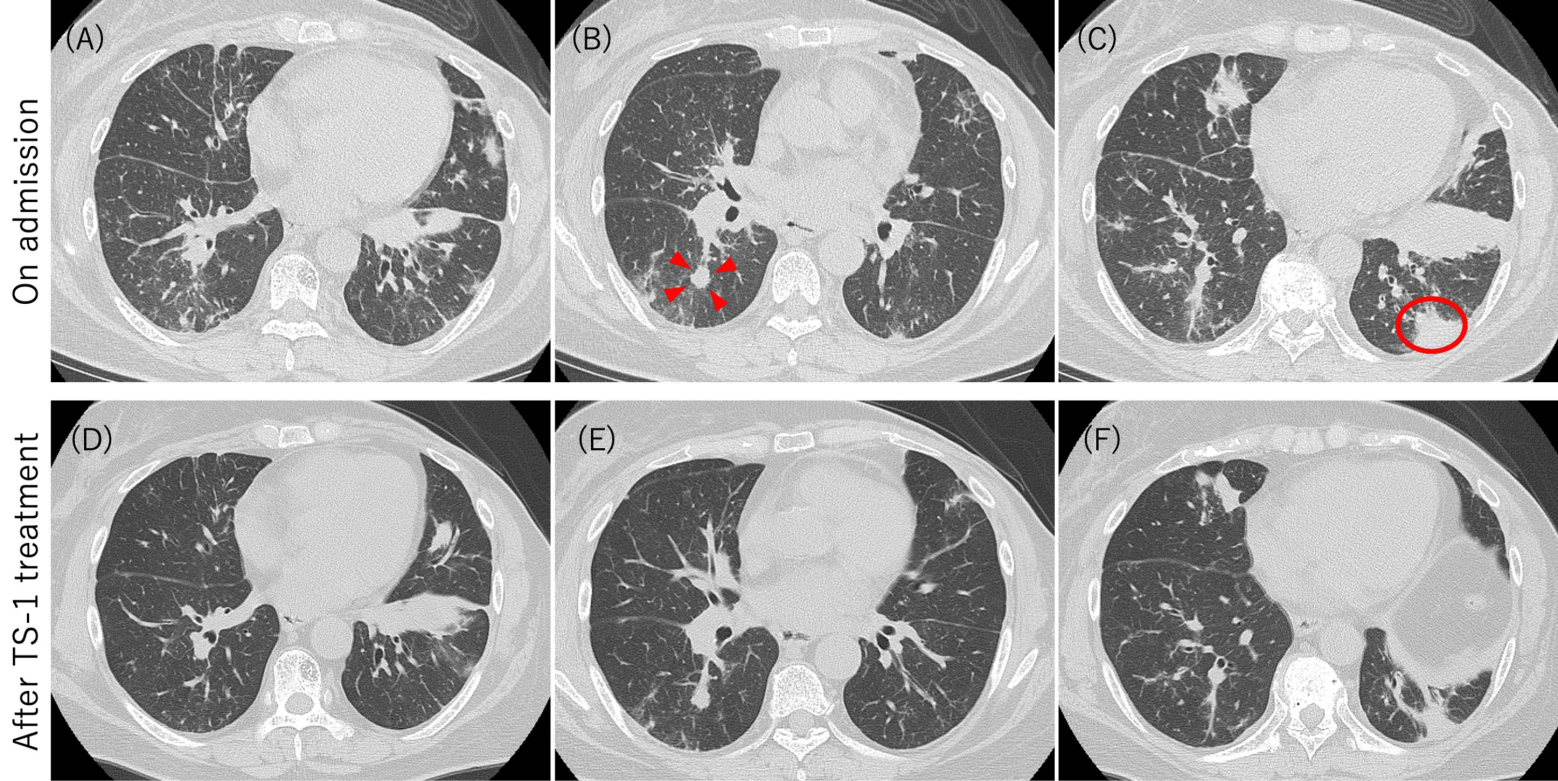
Serial chest CT findings on admission and after S-1 therapy Axial thin-section CT images obtained on admission (A-C) demonstrate multifocal OP-like air-space opacities, including patchy peribronchovascular and subpleural consolidation with surrounding ground-glass changes. Arrowheads in B indicate preexisting metastatic lesions recognized prior to admission. The circled area in C marks the target lesion from which the transbronchial biopsy was performed. Follow-up CT images after S-1 treatment (D-F), at corresponding levels, show marked interval regression of the consolidations and metastatic opacities, with only minimal residual linear or reticular changes. OP, organizing pneumonia

The patient developed dyspnea four months before the current admission. She was referred to the oncology department of our institution three months prior for evaluation of potential eligibility for new therapeutic agents. A surgical biopsy of the metastatic lesion was considered for biomarker analysis. A CT scan one month before admission revealed new bilateral bronchovascular bundle thickening with surrounding GGOs and areas of consolidation, raising suspicion for OP (Figure 1A-1C). She subsequently developed a productive cough and was referred to our Department of Respiratory Medicine for further evaluation.

On admission, her vital signs were as follows: pulse rate 110 beats per minute, blood pressure 159/78 mmHg, body temperature 36.6°C, respiratory rate 18 breaths per minute, and oxygen saturation (SpO₂) 95% on room air. Auscultation revealed diminished breath sounds on the left and rhonchi on the right. Laboratory investigations showed elevated levels of carbohydrate antigen 15-3, while surfactant protein D levels were normal. Other routine blood tests were within normal limits or consistent with her known comorbidities.

Chest CT at the time of referral demonstrated bilateral bronchovascular bundle thickening with surrounding GGOs and areas of consolidation, predominantly in the lower lobes. These findings were superimposed on a previously known pulmonary nodule in the right lower lobe, consistent with metastatic disease. The new infiltrative pattern with peribronchial and perivascular distribution was suggestive of OP, creating a diagnostic dilemma. Notably, the coexistence of preexisting metastatic nodules alongside the new consolidative changes raised the possibility that the OP-like pattern represented an atypical form of disease progression.

Given the clinical history, elevated tumor markers, and diagnostic uncertainty, bronchoscopy was performed. Bronchoalveolar lavage (BAL) and brush cytology were obtained, and a transbronchial biopsy was performed on the left lower lobe. Bacterial cultures from bronchoscopy yielded only commensal organisms (*Fusobacterium* species and *Veillonella* species), and acid-fast bacilli cultures were negative. Brush cytology was Papanicolaou class II (no malignancy), while BAL cytology from the left lower lobe was class V (malignant).

**Figure 2:**
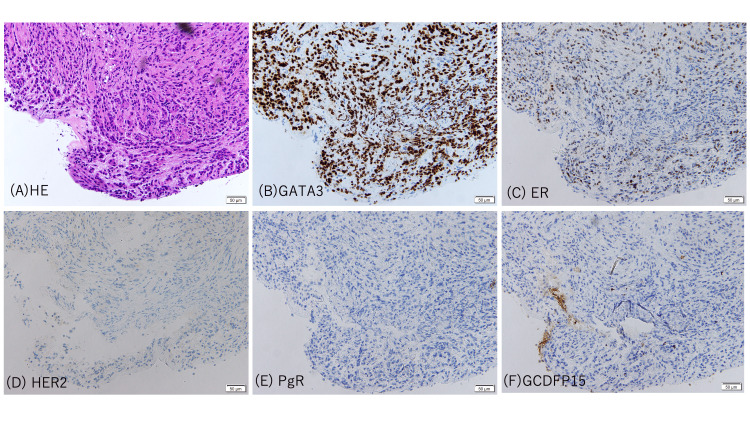
Histopathology and immunohistochemistry of the transbronchial biopsy specimen (A) H&E staining shows infiltrating carcinoma composed of atypical epithelial cells in a fibrous stroma. Immunohistochemistry demonstrates strong nuclear positivity for GATA3 (B), supporting a breast origin. ER shows focal/weak nuclear positivity (C), whereas HER2 (D) and PgR (E) are negative. GCDFP-15 is negative (F). Scale bars: 50 μm. ER, estrogen receptor; GATA3, GATA-binding protein 3; GCDFP-15, gross cystic disease fluid protein-15; HER2, human epidermal growth factor receptor 2; PgR, progesterone receptor

Histopathological examination of the transbronchial biopsy specimen demonstrated adenocarcinoma infiltrating along the alveolar walls. Immunohistochemical analysis revealed positivity for estrogen receptor and GATA-binding protein 3 (GATA3), consistent with a breast primary origin. Gross cystic disease fluid protein-15, human epidermal growth factor receptor 2 (HER2), and progesterone receptor were negative (Figure 2). These findings confirmed the diagnosis of pulmonary metastasis from breast cancer presenting with a consolidation pattern mimicking OP on chest CT.

Given disease progression despite fulvestrant, suggesting endocrine resistance, treatment was switched to S-1 (tegafur/gimeracil/oteracil) at 100 mg/day on a two-week-on, one-week-off schedule. Follow-up chest CT performed four months after initiating S-1 demonstrated significant improvement of the bilateral consolidation and GGOs (Figure 1D-1F), confirming treatment response.

## Discussion

Breast cancer lung metastases classically appear as multiple, well-circumscribed nodules, but atypical patterns such as lymphangitic carcinomatosis, endobronchial disease, cavitation, GGOs, and an air-space (pneumonia-like) pattern have been described [2,3,8]. In the air-space pattern, tumor cells can spread along intact alveolar walls (lepidic growth), producing consolidation with air bronchograms and an OP-like distribution. Importantly, OP-like consolidation caused by metastatic tumors has been attributed to at least two mechanisms: (i) air-space/aerogenous spread with lepidic growth, producing an “air-space pattern” of metastasis that closely mimics OP [9], and (ii) reactive/secondary OP developing in the peritumoral lung [10]. In our case, the biopsy demonstrated carcinoma consistent with metastatic breast cancer without histologic OP, supporting the former mechanism; however, because the specimen was obtained by transbronchial lung biopsy, the sample size was insufficient to fully assess the architectural distribution of tumor cells or to definitively prove aerogenous spread across air spaces. Because this appearance overlaps with OP and infection, imaging alone is often insufficient, and histology is needed to distinguish tumor infiltration from reactive organizing change [2,3,5,6,9].

Only a small number of breast cancer cases with an OP-mimicking pattern have been reported. Prior reports include patients initially treated for bronchiolitis obliterans OP with corticosteroids without improvement, cases suspected to represent drug-induced pneumonitis, and a report in which surgical biopsy initially showed OP without malignancy before subsequent progression and repeat sampling revealed metastatic breast cancer. These cases emphasize that an OP-like radiological pattern does not exclude metastatic disease and that sampling error can occur when tumor involvement is patchy [5-7].

In breast cancer patients, the differential diagnosis for an OP-like CT pattern includes COP, infection, radiation-related OP, drug-induced pneumonitis (including anti-HER2 therapies), and metastatic disease. In our patient, elevated tumor markers without serum inflammatory markers increased suspicion for progression, while bacterial and mycobacterial cultures were negative. Ultimately, transbronchial biopsy with immunohistochemistry was decisive, highlighting that early tissue diagnosis is critical when clinical context and imaging are discordant [4,11-13].

Our case also illustrates clinically relevant aspects of metastatic breast cancer biology. Very late recurrence can occur, particularly in hormone receptor-positive disease, and relapse should not be dismissed solely because of a long interval from the primary tumor [14]. In addition, receptor status can change over time; PgR discordance between primary and metastatic sites is relatively frequent and may reflect clonal evolution under treatment pressure [15,16]. In this setting, lineage markers such as GATA3 can help confirm breast origin when morphology is nonspecific [17].

The radiological response to S-1 provides a clinically useful point. When an OP-like pattern is caused by metastatic disease, corticosteroids alone are unlikely to help, whereas appropriate systemic therapy can lead to rapid improvement. Clinicians should consider metastatic progression in breast cancer patients who develop new OP-like infiltrates and pursue BAL and biopsy promptly to avoid diagnostic delay and inappropriate treatment.

Limitations include the short radiological follow-up after initiating S-1 and the inherent lack of generalizability of a single case. Nevertheless, given the rarity of this imaging presentation, reporting such cases may improve recognition and encourage timely tissue-based diagnosis.

## Conclusions

Metastatic breast cancer can rarely present as OP-like consolidation on chest CT. In patients with a remote history of breast cancer, persistent or unexplained OP-like opacities should prompt early consideration of metastasis and timely tissue confirmation. Bronchoscopic biopsy with immunohistochemistry, interpreted in the clinical and radiologic context, is key to establishing the diagnosis and guiding appropriate treatment.
